# The use and application of intensive care unit diaries: An instrumental multiple case study

**DOI:** 10.1371/journal.pone.0298538

**Published:** 2024-02-29

**Authors:** Maria Johansson, Ingrid Wåhlin, Lennart Magnusson, Elizabeth Hanson

**Affiliations:** 1 Intensive Care Department, County Hospital, Region Kalmar County, Kalmar, Sweden; 2 Department of Health and Caring Sciences, Linnaeus University, Kalmar, Sweden; 3 Research Section, Region Kalmar County, Kalmar, Sweden; 4 Swedish Family Care Competence Centre, Kalmar, Sweden; University Hospital Cologne: Uniklinik Koln, GERMANY

## Abstract

**Aims and objectives:**

The study aim was to explore the use of an Intensive Care Unit (ICU) diary within four different ICUs units in Sweden and thereby contribute to practice guidelines regarding the structure, content and use of an ICU diary.

**Background:**

ICU diaries are used to aid psychological recovery among critical care patients, but differences remain in diary writing both within and across countries. Few studies have focused on the combined views and experiences of ICU patients, family members and nursing staff about the use of ICU diaries.

**Design:**

An instrumental multiple case study design was employed.

**Methods:**

Three focus groups interviews were carried out with 8 former patients and their family members (n = 5) from the research settings. Individual interviews were carried out with 2 patients, a family member and a nurse respectively. Observations, field notes, documentary analysis and conversations with nursing staff were also conducted. Consolidated criteria for reporting qualitative research (COREQ) was followed.

**Results:**

The qualitative findings firstly consisted of a matrix and descriptive text of the four ICU contexts and current practices. This highlighted that there were similarities regarding the aims and objectives of the diaries. However, differences existed across the case study sites about how the ICU diary was developed and implemented. Namely, the use of photographs and when to commence a diary. Second, a thematic analysis of the qualitative data regarding patients’ and family members’ use of the ICU diary, resulted in four themes: i) the diary was used to take in and fully understand the situation; ii) the diary was an opportunity to assimilate warm, personalised and human care; iii) the diary was used to manage existential issues; and iv) the diary was a tool in daily activities.

**Conclusions:**

Analysis of the instrumental case study data led to the identification of core areas for inclusion in ICU diary practice guidelines.

## Introduction

Intensive Care Unit (ICU) diaries are increasingly being used in intensive and critical care nursing practice as an intervention that may facilitate the patient’s psychological recovery. Namely, by describing what happened to them in the ICU and about their health condition [1–3] with the aim of helping to put possible unpleasant memories into context [4]. The authoring of diaries may help both patients and family members process the critical illness experience during and after the time in the ICU [1,5–7] as well as support family members’ bereavement process when the patient does not survive [6–8]. Due to the COVID 19 pandemic, some ICUs opened up fewer diaries because of worries about infection control and due to excessive workload. In addition, visiting restrictions limited the use of diaries written by family members [9]. However, web-based solutions are under development and may in the future become a replacement for or supplement to hard copy diaries [10]. A hard copy diary is described as a handwritten booklet written in collaboration with nurses/staff members and family to the unconscious patient based on a chronology of clinical and social events that occurred during the ICU stay [6]).

## Background

Qualitative studies have highlighted how the written content in the diaries helped patients to realize what they had undergone, provided a sense of coherence and understanding after a critical illness [11–13] and acted as a support in the process of setting up realistic goals for recovery [7,11,14]. According to Backman & Walther [6] if photos are added, they might augment the information and help to make sense of the information provided, akin to a reality check. The diaries could also convey a feeling of humanised care despite being immersed in a technological environment [15].

Earlier quantitative studies have revealed how the diaries reduced the occurrence of patient post-traumatic stress, depression, and anxiety related to critical illness and critical care [16,17] as well as aid the wellbeing of their relatives [18,19] and improved patient health-related quality of life after a critical illness [20].Overall, the quantitative studies have been criticised due to small sample sizes and differences in the diary’s application [21,22]. Thus, it remains unclear as to the efficacy of ICU diaries in reducing PTSD, anxiety and depresession in patients and family members [9]. The majority of previous studies consist of mainly qualitative studies, which highlight that the diary helped to fill in the memory gap of the patients [6,7,23]. Also, the diaries assisted the patient when working through their ICU experiences [14,24] and at the same time provided individual or improved quality care [14,24]. Taken together, considerable diversities remain in diary writing both within and between countries which make it difficult to draw comparisons among the existing empirical studies. A major identified barrier regarding the use of ICU diaries for health care professionals is lack of dedicated time, which was further exacerbated during the COVID-19 pandemic [9]. A consensus exists on the need for guidelines to support diary writing for use in current critical care nursing practice [25–27]. In this regard, the international ICU diary network (established in 2012 by a group of ICU nurses as a non-profit organisation) acts as a vehicle to help with the implementation of ICU diaries via coordinating information, projects and new empirical studies for interested health care staff [28].

## Aim

The overall aim is to explore the use of the ICU diary within four different ICU units in Sweden, and thereby contribute to practice guidelines regarding its structure, content and use.

The research questions were as follows:

1. What are the current practices surrounding the use of a diary in the chosen ICU settings? What local guidelines exist (if any) and in what ways (if any) are they implemented in clinical practice?
2. What strengths or positive aspects are identified by nursing staff, former patients, and family members of ICU patients with regards to current practices in the chosen research settings concerning the use of an ICU diary?
3. Further, what are the challenges or drawbacks that are identified by nursing staff, former patients and family members of ICU patients with regards to current practices in the chosen research settings concerning the use of an ICU diary?
4. What are the core areas for consideration arising from this multiple case study that can be taken up in future development work concerning national clinical practice guidelines on the use of the ICU diary?

## Methods

An instrumental multiple case study design was adopted for the study. An instrumental case study enables a contemporary phenomenon to be investigated in-depth and in its real-world context–in this instance, the use of ICU diaries. Further, a multiple case study approach was chosen as it enabled the researchers to explore similarities and differences in the individuals’ experiences within cases and compare and contrast the experiences across cases [29]. A case study design provides opportunities to use multiple data sources to answer ‘how’ and ‘why’ questions- in this case, about ICU diary use, implementation and how to further develop the diary. The use of several data sources may also enhance data credibility to reach a holistic understanding of the phenomenon [29,30]. The use of evidence from multiple sources also means a kind of triangulation, where the findings have been supported by more than a single source of evidence [28]. This study followed Consolidated criteria for reporting qualitative research (COREQ): a 32-item checklist for interviews and focus groups [31] (See S1 Table).

### Study setting

There are approximately 41070 admissions to 82 adult intensive care units in Sweden annually [32]. ICUs are situated in university, county and district hospitals. County and district hospitals have general ICUs. The ICUs in university hospitals may have a range of specialized ICUs, for example; burns ICU, neurosurgical ICU, medical ICU, surgery ICU and thoracic surgical ICU. The specialized ICUs serve other hospitals in their region when specialized care is required. The study took place in 2018 between March and October at four different Swedish ICUs (units A, B, C and D) in four ICU settings, equal to four cases where the ICU diary has been developed and implemented. Henceforth, ICU settings are called cases, which means everything associated with the settings, such as documents, observations, notes, routines, interviews, and study participants. One ICU setting was a university hospital, and three were county hospitals (See Table 1).

**Table 1:** Characteristics of the participant ICU settings.

Hospital Case	Kind of hospital	Catchment-area People	Kind of ICU	Average length of ICU stay	ICU Admission /year	Number of beds in ICU	Ratio patient/ staff in ICU
**A Hospital**	County	140 000	General	2,58	549	7	1,1
**B Hospital**	University	1 000 000	Thorax ICU	2,09	776	8	1,1
**C Hospital**	County	180 000	General	2,28	634	9	1,1
**D Hospital**	County	149 000	General	2,92	534	7	1,2

The included cases were purposively selected as they were representative of a diversity of care provision, context, and implementation practices of the ICU diary [29]. All participant hospitals were in the same geographical region of Sweden which covers an approximate radius of 170 km by 46954 km^2^, and they all formed part of a regional research collaborative initiative financed by the involved health care regions to stimulate closer cooperation concerning health care research. Nursing staff consisted of specialist critical care nurses with a bachelor level degree and assistant nurses. The staff-patient ratio at the time of the study was between 1.0 and 1.2. Medical staff consisted of 1–2 chief intensive care specialists and several junior physicians and at the units there were 1–2 head nurses of the department. There were no psychologists attached to the ICU units. Nurses, physicians, and nurse assistants provided psychological support to patients and their family members. ICU diaries have been used at the participating units for between 25–30 years.

### Data collection

A protocol for conducting a multiple case study was established (See S2 Table) in accordance with Yin’s (29). The protocol was a procedural guide for collecting data for a case, including a set of field questions to be addressed by the researcher (first author, MJ). The role of the protocol was also a standardised agenda for the researcher’s line of inquiry to increase reliability [29]. The data sources included observations, field notes, documents, informal, unstructured field interviews, a telephone interview, individual interviews, focus group interviews and a text message. A trusted external secretary with prior experience as a medical secretary transcribed the recordings.

#### Observations

The first author (MJ) engaged in direct observations of selected cases for approximately 24–32 hours respectively in the ICUs (B, C, D). MJ was working clinically at that time in ICU A, and was subsequently familiar with the unit thus needed no assistance in collecting documents.

#### Field notes

Field notes consisted of a continuous record of responses reported from the different units according to the study protocol. They were written by MJ both in the ICU but also directly after each observation period.

#### Documents

Collected documents included pamphlets related to the patient in critical care, documents about patient diary guidelines and informed consent forms about diary writing/photography.

#### Informal field interviews

The field interviews took the form of spontaneous, informal conversations which described the nursing staff’s views and experiences of how the ICU diary was used within everyday nursing practice on their respective unit. These informal interviews were not audio-taped but rather written up in the form of field notes by the first author (MJ).

#### Individual interviews

An interview was conducted with one patient and their family members recruited via telephone from the ICU follow-up programme at one of the ICU settings. The interview was based on the questions from the study protocol but reformulated into open-ended questions (S2 Table). Moreover, an interview with a nurse with experience of developing diary implementation in Sweden was carried out in the same way. The interviews were audio-taped with the participants’ permission, ranging from 60–70 minutes in duration and subsequently transcribed verbatim. These participants were originally invited to the focus group interviews but were prevented from attending for various reasons. Nevertheless, they wished to communicate their experience of using the diary. A telephone interview/conversation and a textual message from a former patient were also included in the data. Field notes were duly written directly after the telephone conversation had taken place.

#### Focus group interviews

Three focus group interviews with a total of 8 patients and 5 family members at the participant hospitals were conducted to explore the use of an ICU diary in the respective care settings. The design of the ICU diary was developed in keeping with each unit’s guidelines and practices. The participants were only aware of the version they had received from their respective ICU. Nurses from the individual ICU follow-up programme recruited the participants via telephone. Information about the focus group sample is provided in S3 Table.

Patients had been discharged for a period of seven to fourteen months depending on their recovery process and were invited to bring family members with them to the focus group session. The inclusion criteria for patients and family members included; being a former ICU patient, having an ICU diary, being willing to share their experiences and aged 18 years or older.

In accordance with Krueger & Casey [33], open-ended questions were posed in order to allow for explanations, descriptions and illustrations relating to ICU diary writing and practices. Key questions included the following; how was it to read/write in the diary? When was the diary handed over? When were the photos handed over? What are your thoughts about the content in the diary? MJ moderated all focus group interviews. The second author (IW), a critical care nurse specialist with a PhD in caring sciences, operated as an assistant moderator and concluded the interview by briefly summarizing the main points and asked if the summary reflected what participants had experienced in the group [33]. Interviews were audiotaped, and were of 86–127 minutes in duration. The data were transcribed verbatim. Unstructured field notes were written by MJ directly after the interviews to document key remarks and reflections, such as something surprising and/or unexpected to MJ or how a group’s responses were similar to or different from earlier focus group sessions [33].

### Data analysis

In keeping with Yin’s [29] recommendation with case study analysis work, the authors duly returned to the core research questions. To help answer the first research question, the extensive amount of practical information from the observational studies, documents and informal, and unstructured field interviews were systematised with the help of a matrix (see Table 2 below) developed from the study protocol (See S2 Table) [29].

**Table 2:** Matrix of current ICU diary practices in the four study settings.

Hospital Case	Case A (K)	Case B (L)	Case C (N)	Case D (J)
**Guidelines including practices surrounding the use of a diary**	Oral	Oral	Written	Oral
**Voluntary writing**	Yes	Yes	Yes	Yes
**Format**	Physical A5 format	Physical A5 format	Physical A5 format	Physical A5 format
**Standard headings in the diary**	No	No	No	No
**Introduction and aim in the diary**	Yes	Yes	Yes	Yes
**A page where Family members informed about habits**	Yes	Yes	Yes	Yes
**Structure with summary, daily entries and end note**	Yes	Yes	Yes	Yes
**Glossary in the diary**	Yes	Yes	Yes	Yes
**Additional content**	No	No	No	Poem at the end of the diary
**Authors**	Nurses, assistant nurses, family members	Nurses, assistant nurses, family members	Nurses, assistant nurses, family members	Nurses, assistant nurses, family members
**Opening up of diary is documented in medical journal**	Yes	Yes	Yes	Yes
**Document regarding consent diary/photo**	No	No	Yes	No
**Account of kept diaries**	No	No	Yes	Yes
**Ownership**	Patient	Patient	Patient	Patient
**Storage of photographs**	No	No	Yes	Yes
**Generic photos of equipment**	No	Yes	Yes	Yes
**Including the photographs**	Immediately	Immediately	At follow-up service	At follow-up service
**Handover the diary**	Follow the patient/family members	Follow the patient/family members	Follow the patient/family members	Follow the patient/family members
**Follow-up service**	Yes	Yes	Yes	Yes
**Responsible group for diaries**	Yes	Yes	Yes	Yes
**Feedback to the ICU staff**	Yes	Yes	Yes	Yes

Second, to answer the remaining research questions (2, 3 and 4), a thematic analysis, inspired by Braun and Clark [34], was conducted of the qualitative data from the focus group interviews, individual interviews, telephone interview, and textual message [34]. This approach was deemed suitable for identifying, reporting and interpreting patterns of meaning within the transcribed data. It was also chosen for being a flexible yet rigorous method for examining the perspectives of different research participants, highlighting similarities and differences and also generating unanticipated insights [34].

**Fig 1:**
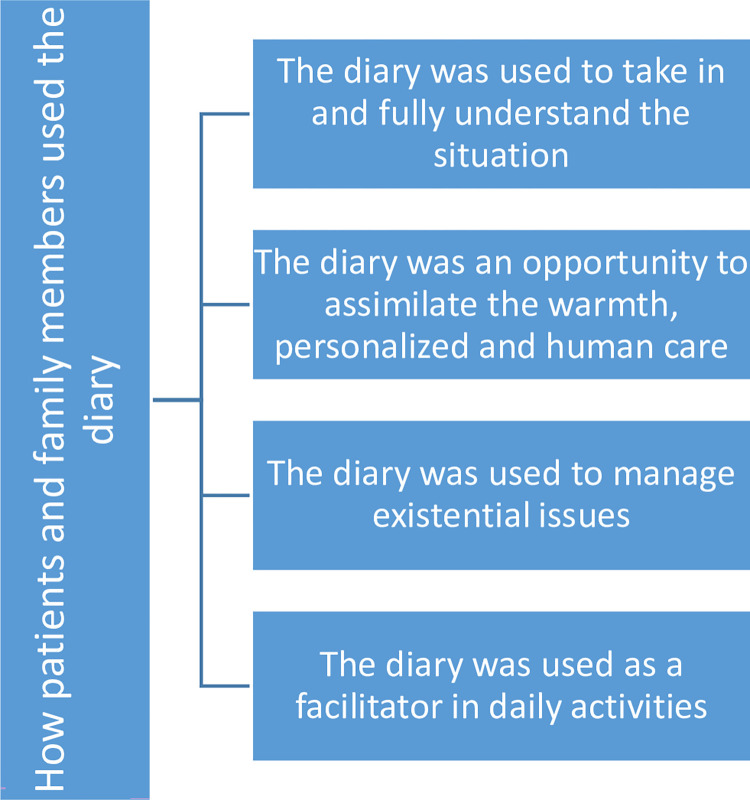
Core themes from the qualitative analysis of interview findings and text message of participant patients and their family members.

The analysis commenced with repeated reading to be familiar with all aspects of the data. Thereafter, a coding process began using highlighters and writing notes on the texts. Extracts that demonstrated each code were brought together in Word documents, one for each pattern. An extract might be uncoded, coded once, or coded many times as it was relevant. This way, all the codes with their extracts were organised in their document. The documents were organised into theme piles. In the final step of the analysis, core themes were developed that reflected patients’ and family members’ use of the ICU diary (See Fig 1 below).

### Ethical considerations

The study was conducted in compliance with the established ethical guidelines of the Declaration of Helsinki [35] and the Regional Ethical Committee in Linköping, Sweden provided formal ethical approval for the study (Dnr 2017/550-31). The clinical medical director at each of the respective units was informed about the study and approved the study. Potential focus group participants were recruited through the respective ICU’s follow-up services, and the hospital with the strategically optimal location was chosen for the interviews. Participants were financially compensated for their travel costs.

Information about the study was repeated verbally to participants prior to the start of the observation study and the interviews. Voluntary participation, informed consent, confidential treatment of all data and the right of participants to withdraw at any time without repercussion to their care or working situation (as appropriate) and appropriate storage of the qualitative data were all emphasised. Informed oral consent by the observation participants was witnessed by the first author and consisted of the participants verbally stating that they agreed to the first author observing activities and interactions involving the participant as part of her research study. Informed oral consent by the interview participants was also witnessed by the first author and consisted of the interview participants verbally agreeing to take part in an interview as part of the first author’s research study. Verbal consents were documented by the first author as part of her methodological field notes and as approved by the regional ethics committee. As the first author was working as a critical care nurse in ICU A, qualitative interviews were not carried out in this particular setting [36].

## Results

Table 2 below provides an overview matrix of the main similarities and differences regarding the structure, content, and ICU diary use within participating ICU settings arising from the systematized observational study, documentary and informal field interview data as outlined above. The matrix is supplemented with a written summary description of the four ICU contexts and main current practices drawn from the qualitative data. Core findings from a thematic analysis of the qualitative data are subsequently presented.

### ICU contexts

It was self-evident by nursing staff in all ICUs to involve family members in writing/reading the diary without them feeling obliged to write. Family members might come and go around the clock at some units, but in others, staff agreed on their visiting times. ICU nursing staff had diverse experiences of diary writing from 10 to 23 years. Prepared diaries were readily available on the ICUs in a room intended for follow-up services.

Staff from the general ICUs’ follow-up services had regularly networked since 2012, together with another two ICUs in the region in order to exchange experiences about diary implementation and follow-up services. At that time, all participant ICUs had the same members in the follow-up services and in the diary group who prepared and were responsible for diary implementation.

### Guidelines

Nursing staff at each ICU had established their own purpose statement in the absence of national guidelines on patient diaries. Diary writing had been realised as a spontaneous initiative by nursing staff, and the activity had advanced often through “learning by doing”. The assumption being that the patient needed to know and come to terms with what happened while they were in ICU. Also, family members needed help to also understand and process the patient’s stay.

Written guidelines were found in one ICU and comprised inclusion, exclusion criteria, writing style, informed consent, the process around photographing, and handover of the diaries. Another unit was developing written guidelines, but the other units had only oral guidelines about the structure, content, and use of the diary. Experienced nursing staff communicated informal guidelines to new employees and less experienced ICU nursing staff. The absence of clear, written guidelines meant that many patients did not receive a diary because of the hesitation in deciding which patients should get a diary and how to prioritise the process. The uncertainty was expressed as a cause of frustration among the participant nursing staff.

#### Diary structure

The diary was an A5 notebook, lined, laminated cardboard folder, and spiral bounded or bound. Some ICU nursing staff had chosen a picture of a well-known sight from the town to act as the cover. Others had chosen colourful images such as flowers or trees. The ICU diaries’ format was open, only blank pages, handwritten, and without any editing (that is, there was no one responsible who approved the diary before it was handed over to the patient). On the initial page/s, the diary included dedication to the patient and the patient’s name.

Next, diaries usually contained a summary and a reason for the patient’s admission to ICU and daily entries. The summary and closing framed the diary’s story about the time in ICU. There was also a glossary, and in three of four ICUs, generic photos on the most common ICU medical equipment.

### Diary content

All diaries contained a page called ‘Who am I? What is important for me?’ The page asked for the patient’s sleep habits, favourite sports, favourite food and drinks, music, and philosophy on life which family members were requested to complete. The diaries in some ICUs comprised the 24-hour circle in the ICU with an associated timeframe. This information highlighted that the activities varied around the clock and explained that somebody was always nearby the patient. Next followed the narratives of daily activities with and around the critically ill patient, which formed a central part of the diary. The narratives contained information about medical treatment, daily care of the patient, information about progression, such as mobilising out of bed, conversely how the patient’s condition had deteriorated, and that the patient was approaching death.

Other entries were psychosocial, which expressed and confirmed the presence of the family members at the bedside. Family members added information about what happened at home or greetings from other family members. Entries included reflections around the weather, seasons, Swedish festivals, sports events together with entries of a more personal nature. Staff members who wrote the entry included the whole team, dated, and signed the note. A unique contribution from staff at one ICU was a poem at the end of the diary intended to provide a respectful content if diary entries were sparse.

### Use of the diary

All units kept the diaries at the patient’s bedside so that nursing staff and family members could write whenever they wanted to. The diary was seen as the patient’s property and was not deemed to be part of the patient’s medical records. Written or oral guidelines stated that a diary should be offered to the patient when they were expected to be cared for >72 hours or for those who ‘needed’ it. In one ICU, there was an ambition to open up a diary to all patients admitted to the ICU, especially patients cared for following a cardiac arrest, which meant a shorter care stay than three days. Care of patients with cardiac arrests denoted heavy sedation, ventilation and connection to a range of cannulas and tubes for about 24–36 hours and then awoken. In general, this group were deemed to benefit from a diary.

The nursing staff did not open an ICU diary for patients with severe brain injuries, and dementia, poor prognosis nor for patients who did not speak/understand Swedish. Often, some nursing staff commenced a diary immediately when a patient was admitted to ICU, but sometimes days elapsed, and the diary writing did not take place. Reason/s for the delay included lack of time or difficulties to start the diary as the summary was deemed to be the most essential and extensive aspect. In some units, assistant nurses frequently opened a diary as they had more time to initiate the process than the registered nurses.

Two ICUs kept registers about patients who had a diary. This included a checklist with the patient’s name and informed consent given by family members to photograph the patient. The checklist was stored in a diary file in the staff room and accessed by nursing staff who belonged to the diary group. Other ICUs asked family members for informed consent orally in conjunction with information about the diary without any written documentation. The consensus was that the photographing was a part of the treatment and therefore did not necessitate permission from the patient or family members, thereby facilitating the prompt starting of a diary and with photographs. However, the patient was required to give retrospective consent once they were sufficiently well enough to receive the diary.

### Use of photographs

All staff from the four ICUs used photos to augment the written content. Nursing staff were aware of the photos’ supportive role and that the patients frequently requested more pictures. Usually, the first photographs were taken when the patient was fully sedated, ventilated, and linked to a range of tubes and cannulas. Subsequent photos were taken on significant events and progressions during the ICU stay. Typically, the patient was photographed at a distance so as not to expose the patient at an inappropriate angle.

However, nursing staff at one ICU meant that the pictures should be realistic, and nursing staff should go close to the patient when photographing because the patient needed to recognise themself. The photos should assist as a ‘reality check’ when setting goals for recovery.

Three ICUs used a digital camera with memory, where nursing staff might print out the photograph straightaway and then delete the original from the memory card. The fourth ICU moved and stored the pictures in image management on a desktop. At one ICU, the pictures were pasted in the diary immediately, close to the written text, so that text and image interacted. Sometimes, photos were collected in an envelope at the back of the diary.

Another ICU used mounted the photos directly in their context in the diary but left the other side empty. This made it possible for the patient to remove the picture but without destroying any existing text.

In contrast, two ICUs did not include the photos directly in the diaries. The rationale being that the patient needed a ‘face to face’ meeting with ICU nursing staff and an explanation of the photos to fully understand the pictures and be allowed to ask any questions. Otherwise, they feared that the patient might be frightened or re-traumatised if no explanations was given. Another reason for the personal meeting was to obtain retrospective consent for the pictures. If the patient refused the photos, they were destroyed.

### Handover process

The diary followed the patient by the nursing staff placing the diary at the end of the bed or giving it to family members on transfer to the general ward. Diary group/follow-up service staff visited the patient at the ward when they were transferred from the ICU. During this visit, the photographs were discussed in those cases they were not included in the diary. If the patient wanted the photos at that time, they were handed over. Otherwise, the pictures were discussed at the follow-up service later and then handed over. Patients who did not attend any follow up service received their photos by mail at home, if they requested them.Family members where the patient did not survive, received a telephone call about 6–8 weeks after the patient’ death and were invited to the follow-up service to discuss the patient’s stay at the ICU.

### Thematic analysis findings

Four interconnected themes were derived from the qualitative thematic analysis that reflected patients’ and family members’ use of the ICU diary. The themes are represented in the following sections and highlighted in Fig 1 below. Participants are cited using the number of the focus group and P (atient), S (pouse) number identifier.

#### The diary was used to take in and fully understand the situation

The chronology in the diary and the everyday language were understood as valuable characteristics because family members expressed feeling unsure about specific dates and events during their family member’s ICU stay. Even in the diary, patients and family members sometimes had difficulty distinguishing specific dates. A positive aspect of the ICU diary highlighted by participants was the ability to read and re-read the information to assimilate it.

*That you can go and look in the diary a little now and then and think about it* (*I/S1)*

Patients and family members requested ongoing summaries in the diary, as the information was sometimes perceived to be insufficient or irregularly provided, especially during more extended periods of stay. The diary was mainly experienced as a tool to help process the time in ICU by describing what daily life looked like, which was not included in the medical records.

Patients described dreams, nightmares and unreal experiences that caused long-standing problems post-ICU discharge despite a few other more positive memories. This meant that patients and family members had different experiences of events which sometimes led to misunderstandings among families. However, patients expressed missing entries with sufficient personal details which described confused thoughts they had expressed and what the thoughts were about, so they might orientate themselves. Patients expressed a need to know that these confused thoughts were often of a frequent occurrence.

*The nursing staff explained to my wife and children–but nothing in the diary*, *about the confused thoughts*. *They said it calmly*, *and that he’s going to recognize you*. (*II/P3)*.

All patients and family members emphasized how important the summary was, which told the story prior to admission and why the ICU admission had taken place. Despite this, patients and their family members experienced that critical and life-threatening episodes were sometimes neglected, and they wanted more realistic adjectives to be used such as “deadly condition” and “hovering between life and death” to understand how critically ill the patient was. Another drawback relating to missing information was the setting’s description with its sounds, sights, machines and bed. In other words, former patients wanted to have events clarified, which were known to be the source of nightmares, distorted thoughts and hallucinations; as an example, the ICU bed was often experienced as a container, boat or a railway truck. The ICU diary became a way of summarizing and making countless details visible in a single concrete picture. It was considered as a reliable document against which to check their personal memories of their ICU stay.

*The diary is fantastic; without it*, *I would not have known anything*. *The wife has a hard time reading the diary*. *The children also find it difficult*. (*I/P2)*.

The photographs were expressed by patients as being personal and they offered them a window into the past to fully understand their history. All insisted that it was important that photos were tightly connected to the written texts. Some had received photos in bulk (in envelopes) connected with the follow-up services, to insert them independently in the diary.

*I had a need to get a whole of the ICU stay*. *I got loose pictures that I could not put into context* (*telephone interview)*

In general, patients wanted realistic photographs without embellishments because they wanted to know their actual health status in order to set realistic rehabilitation goals. They also requested close-ups to be able to recognize themselves, otherwise, they argued that it could be anyone in the photo. Patients with realistic pictures of themselves with all the equipment, expressed that it did not feel so scary anymore.

*Yes*, *but then that’s not pleasant*, *you see it’s me*, *but further away*, *then it wouldn’t have been easy to see that it was me* (*II/P2)*.

#### The diary was an opportunity to assimilate the warmth, personalized and human care

Patients and family members found the diary to be personally touching. They explained that naming the patient in each entry gave identity and a personal touch to the diary. They found an unexpected kindness in the staff’s diary writing which deeply moved them. The diary was thoughtful and often well-written with warmth that patients expressed that they would carry with them for life.

*They (nursing staff) really showed from the bottom of their hearts that they liked me*, *that I’m important* (*II/P2)*.

The diary bore traces of the authors’ varying ability to express themselves, which participant patients and family members appreciated. Family members felt that the nursing staff was there for them all the time, even if it was for the patient’s sake. The diary testified that the patient was cared for with respect and not as an object. For family members, the diary was considered a symbol of human care and hope in situations where the outcome for the patient was uncertain during the ICU stay.

*We can read in the diary*. *‘We see a slight improvement’*. *This little word is so important* (*I/S2)*

Some former patients proudly read descriptions such as “the ward’s trump card”, “the ward’s sunshine story”, which further underscored the personalized caring and how sick the patient had been and survived the trauma. Others read about how the nursing staff washed the hair of unconscious patients, helped with the first shower, provided offers of TV watching and fulfilled requests for particular drinks.

*I think it’s nice to read*. *Today you have sat on the edge of the bed*, *and it’s the nursing staff who wrote*. *They (nursing staff) care so much about one*. *It warms*. (*I P3)*.

Patients emphasized the importance of the family members’ notes. However, many family members admitted that they could not write in the diary themselves. They expressed that they were too sad and worried, but they experienced it was good to read the nursing staff’s entries as they found it to be sign of hope among all the sorrow. Patients felt that family members had been well-taken care of. Patients and family members expressed that the support and empathy that they could make out in the diary acted as a long-standing form of support for them.

#### The diary was used to manage existential issues

Spiritual issues about the meaning of life emerged for patients experiencing a life-threatening illness, especially when the trauma was lived through and vividly described in the diary. Some of the patients had experienced a near death experience on a visit related to cardiac arrest and asked themselves. Why did I get sick? Why did I survive? The patients might go back and look in the diary from time to time and tend to ask themselves the same existential questions. Patients admitted that it was often serious issues that were raised and that were difficult to share with “outsiders”. Patients and family members believed that one must have been there to fully understand. The pictures in the diary might explain the problematic situation and the ever-present threat of death. They explained that the diary “sharpened the mind”, to pay intention to how fragile life could be and how quickly it could change.

*However*, *it was strange to me; why did I get this disease*. *I kept thinking*, *why did I have a cardiac arrest*? *For twenty years I haven´t had anything*, *but suddenly I got it* (*II/P2)*.

Family members admitted that it caused a disturbance in the family, when a member was ill with a life-threatening illness but that the family was brought together by reading the diary. Some patients noted that they found it challenging yet exciting to read what they had been through in the diary and experienced gratitude that they were alive and believed that life was now too short to dwell on existential issues. Patients admitted that they were not afraid of the future and felt that the experience had empowered them in a way as they now appreciated life even more. Even for family members, existential issues could deeply touch them as illustrated by the following quotation.

*Mum*, *where do you have the diary*? *So*, *she can crawl away for herself*, *so she is 27*, *and then I see how a few tears fall*, *and then she comes and holds me*, *says mother*, *I’m so glad you’re here* (*II/P3)*.

#### The diary was used as a tool in daily activities

Family members described the diary as a tool for information and communication during the ICU stay. To access, understand and take in the medical information. They found the diary glossary to be useful as it often explained the use of medical terms, which eased conversations with nursing staff and provided a feeling of being included. Family members described the diary as available when needed and that they used the diary as a company, comfort and sometimes as an intimate friend at the ICU.

*It was great to not just sit alone in the waiting room spending time and biting nails*. *It was great to have a diary available* (*III/S1)*.

The diary was viewed by family members as something to keep them occupied with instead of constantly following the monitoring screens and as a way of overcoming uncertainty. Family admitted that they wrote not only for the sick person but also for their own sake to unload their personal worries and thoughts.

On discharge home from hospital, participants explained that the diary became a basis for the patient to ask questions. The diary also became a support for family members to be able to account for the course of events. The diary was perceived as a reliable document about the time spent in ICU. Pictures of family members showed that they had been there during the critical illness.

*All visits are listed here*. *I think it is vital that all the sons are in the pictures and not just S and I* (*I/S2)*.

However, the diary could evoke unpleasant memories for some patients, which caused the diary to be set aside.

*I experienced the diary as a bit silly initially*, *but now I can get hurt by it when I look at it; the trauma remains and gets even more challenging*. (I/P2)

A participant former patient had used the pictures in the diary as proof of how ill they had been in their effort to convince persons in authority. Others had let family members and friends read the diary themselves, as they had not been able to tell and re-tell their story.

## Discussion

The aim of the study was to explore the use of the ICU diary within four different ICUs units in Sweden and thereby contribute to practice guidelines regarding its structure, content and use. The practice recommendations arising from the study findings can be summarised into three areas, namely the need for guidelines, guidelines outlining the content of the diary, and how the diary making process reflected the approach of person-centred care. Each will be considered in turn. This is followed by a discussion of the study’s main methodological considerations.

### The need for practical guidelines

The study revealed that it was left to the discretion of the nurse caring for the patient if a diary was opened up. The rather haphazard selection of patients for keeping a diary has previously been demonstrated in Scandinavian surveys 1 [4,24]. National guidelines could act as a support for priority setting and to provide guidance as to whom and when to open up an ICU diary. A common standard would make it possible to follow up on the diary as a caring intervention continuously and systematically. National guidelines may pay attention to the diary making process and so increase awareness among nursing staff that the diary can act as a useful debriefing tool and as a help in reorientation for the patients [1,2,17,23].

Based on the study findings, family members’ roles in the ICU setting are important to highlight in the national guidelines, including the promotion and support of their active participation in the writing, including the start-up of the diary. It is known from previous studies that family members benefit from the diary [5–7] and that the diary may also act as bereavement support if the patient should die [8].

In the current study, patients with severe critical illness, (little chance of survival), dementia, and people with learning disabilities were automatically excluded. Therefore, guidelines need to outline that the diary may also benefit family members and then the green light is given to commence a diary to broader groups of patients. Further, it is regarded as a human right to be provided equal treatment as a Swedish inhabitant, and this approach would also include patients with other languages [37]. The diary may be translated in some way and/or compiled with more pictures and generic pictures.

The study highlighted that guidelines need to be flexible, only stating certain main principles and being considered for every person admitted to the ICU. Further, it showed how practical knowledge sustained the writing as patient categories cared for shorter periods than three days were included. An example is patients having had a cardiac arrest, who were deemed to need follow-up support as this group of patients tended to develop both cognitive and emotional difficulties [38].

### Guidelines outlining the content of the diary

The study revealed that patients and their families requested relevant, realistic content with close-up pictures in the diary because they wanted to know the main details to gain a better understanding of the patient’s illness and what happened to them during their ICU stay. The findings were contradictory to those of the nursing staff, in keeping with a previous study that demonstrated how the staff weighed every word to avoid harming the patient in any way [39]. It has been previously noted how nursing staff experienced diary writing as “complicated in its simplicity”, due to difficulties finding “the right words” in writing and at the same time providing comfort and confidence that patients and family members needed both during and post the ICU stay [40]. Further, it was noted that pictures were often taken at a distance and avoided being taken of a patient with a swollen face, which is in line with previous findings [14,26]. This poses the question of whether nursing staff are overly cautious with regards to diary content to avoid causing the patient any potential offence or distress. In addition to the diary content.

The process of photographing highlighted the main differences between the four cases and were related to local legal and ethical considerations. Previous research highlighted that photography caused additional considerations and units removed pictures in the diaries that were taken without prior consent and that could potentially impinge on patient privacy [12,14]. On the other hand, patients who were provided photographs appreciated them and wanted additional ones [41]. Previous studies have demonstrated the value of pictures noting that photographs added realism and neutralized frightening fantasies [42] and that the diary with its text and photos can be seen to be important to induce post-experience reflections [43].

### How the diary making process reflected the approach of person-centred care

The participant family members in the study underscored how they were naturally offered to participate actively in care by reading/writing in the diary. In this way, they experienced themselves as valuable and equal partners. Writing the diaries was about doing an intervention with patients and their families in partnership rather than ‘to’ them. Family members highlighted the personality of the critically ill patient, and together with the nursing staff, they helped to meet the patient’s needs for information and support. Writing a personal diary is in line with current healthcare policy concerning people-centred care, which means putting patients and their families at the centre of decisions and working alongside them to secure the best possible outcome [44]. Establishing a partnership in the ICU was challenging because the patient was not awake and unable to tell their life story. However, the diary and the page with questions ‘Who am I? What is important to me? were created to understand the patient as a human being with physical, psychological, and existential needs. This information was often captured from family members and used to tailor conversation topics, activities and motivational features in daily nursing care. Likewise, the diary itself may be regarded as documentation of a piece of life that would otherwise have been unknown to the patient.

Patients expressed feeling cared for when they read a diary written primarily for them as the diaries were dedicated to the patient. The writing helped to maintain a feeling of togetherness among the family members and nursing staff, which has been expressed in previous studies [8,45,46]. The belief that diaries are a caring activity was coined by Roulin, Hurst & Spirig [47] as writers from different backgrounds took part in an everyday activity for the patient’s benefit. A personal diary with personal photographs also demonstrates that the nursing staff has been willing to go that extra mile for the patient [12]. Diary writing also created a caring relationship between the patient, family members and nursing staff [48].

### Methodological considerations

The study was carried out by using a qualitative multiple case study methodology, where the study protocol acted as a guide helping the first author in carrying out the data collection from a single case as one of several in the multiple-case study approach [29]. It can also be seen as a way of increasing the reliability of the case study research and a possibility to replicate findings across cases. Multiple methods of data collection, for example, the combination of focus groups, the individual interviews and notes from observational studies were used to identify similar, different, or complementary views and experiences across the different participant groups. The approach allowed for methodological triangulation and increased the likelihood that findings were credible [29,30].

#### Limitations

A study limitation is that the four ICU settings were located in one geographical area of Sweden so that the degree of transferability of the findings to other settings can be questioned. Nevertheless, the study likely reflects the different use of ICU diaries in Sweden. Further, it can be argued that the findings are applicable to other ICU settings with similar publicly financed health care systems. The study can be considered as a first step in a longer-term research and practice development work towards national guidelines regarding the use of the ICU diary.

The first author (MJ) was an ICU nurse at one of the participating clinics, so it could be argued that she potentially lacked objectivity and was biased in her data collection and analysis activities due to her insider knowledge of ICU A [36]. Nevertheless, MJ kept detailed field notes which included reflections of her researcher role in the settings which were duly discussed on a regular basis with her doctoral supervisors. Also, it can be argued that MJ’s insider knowledge was an advantage as she was familiar with the ICU context so that she was also able to ask relevant questions and was able to have an in-depth understanding of participants’ responses and actions.

## Conclusion

Based on the findings it can be argued that clinical practice guidelines concerning ICU diaries would help to ensure their wider and more consistent use for all ICU patients, as opposed to a more selective groups of ICU patients. Second, they would help to make the role of family members more transparent and valued. Finally, they would provide concrete guidance to nursing staff about how best to write in the ICU diary to ensure relevant, real-world content bringing the human element and aiding with genuine person-centered care.

## Relevance to clinical practice

The findings are intended to act as an impetus for future practice development work concerning ICU diary guidelines in ICU settings in other health care regions in Sweden and internationally in countries with a similar health care system to that of Sweden. The aim being to help facilitate and promote nursing staff’s everyday clinical practices concerning ICU diary writing in keeping with a person-centred approach.

## Supporting information

S1 Table Consolidated criteria for reporting qualitative research (COREQ): A 32-item checklist for interviews and focus groups. (PDF)

S2 Table Study protocol for instrumental multiple case study. (PDF)

S3 Table Description of focus group interview participants. (PDF)
