# Supplementary material for: The use and application of intensive care unit diaries: An instrumental multiple case study
Source: PLoS One. 2024 Feb 29;19(2):e0298538. doi: 10.1371/journal.pone.0298538 (PMC10903823; doi:10.1371/journal.pone.0298538)
Supplement: S1 Table — (PDF) [file pone.0298538.s001.pdf]

S1 Table Checklist COREQ

COREQ (COnsolidated criteria for REporting Qualitative research) Checklist

A checklist of items that should be included in reports of qualitative research. You must report the page number in your manuscript where you consider each of the items listed in this checklist. If you have not included this information, either revise your manuscript accordingly before submitting or note N/A.

**Topic**	**Item No.**	**Guide Questions/Description**	**Reported on Page No.**
**Domain 1: Research team and reflexivity**			
Personal characteristics			
Interviewer/facilitator	1	Which author/s conducted the interview or focus group?	Page 8,9, Title page
Credentials	2	What were the researcher's credentials? E.g. PhD, MD	Cover letter, Title page
Occupation	3	What was their occupation at the time of the study?	Title page, 11, 31, Cover letter
Gender	4	Was the researcher male or female?	Title page
Experience and training	5	What experience or training did the researcher have?	Title page, 9
Relationship with participants			
Relationship established	6	Was a relationship established prior to study commencement?	Page 8, 10
Participant knowledge of the interviewer	7	What did the participants know about the researcher? e.g. personal goals, reasons for doing the research	Page 10
Interviewer characteristics	8	What characteristics were reported about the inter viewer/facilitator? e.g. Bias, assumptions, reasons and interests in the research topic	Page 8, 9, 31
**Domain 2: Study design**			
Theoretical framework			
Methodological orientation and Theory	9	What methodological orientation was stated to underpin the study? e.g. grounded theory, discourse analysis, ethnography, phenomenology, content analysis	Page 4, 5, 9
Participant selection			
Sampling	10	How were participants selected? e.g. purposive, convenience, consecutive, snowball	Page 5, 6, 7
Method of approach	11	How were participants approached? e.g. face-to-face, telephone, mail, email	Page 6 , 7, 8, 9
Sample size	12	How many participants were in the study?	Page 5, 8, S3. Table.

Non-participation	13	Non-participation 13 How many people refused to participate or dropped out? Reasons?	Page 8
**Topic**	**Item No.**	**Guide Questions/Description**	**Reported on Page No.**
Setting			
Setting of data collection	14	Where was the data collected? e.g. home, clinic, workplace	Page 5, 6, 7, 8, 9, Table 1.
Presence of non-participants	15	Was anyone else present besides the participants and researchers?	Page 7, 9
Description of sample	16	What are the important characteristics of the sample? e.g. demographic data, date	S3, Page 5, 6, 7, 8
Data collection			
Interview guide	17	Were questions, prompts, guides provided by the authors? Was it pilot tested?	Page 6, 7, 8, 9, S 2 Table
Repeat interviews	18	Were repeat inter views carried out? If yes, how many?	Page 8
Audio/visual recording	19	Did the research use audio or visual recording to collect the data?	Page 7, 8, 9
Field notes	20	Were field notes made during and/or after the interview or focus group?	Page 7, 8, 9
Duration	21	What was the duration of the inter views or focus group?	Page 9
Data saturation	22	Was data saturation discussed?	Page 10
Transcripts returned	23	Were transcripts returned to participants for comment and/or correction	N/A
**Domain 3: analysis and findings**			
Data analysis			
Number of data coders	**24**	How many data coders coded the data?	Page 10
Description of the coding tree	**25**	Did authors provide a description of the coding tree?	N/A
Derivation of themes	**26**	Were themes identified in advance or derived from the data?	Page 10
Software	**27**	What software, if applicable, was used to manage the data?	N/A
Participant checking	**28**	Did participants provide feedback on the findings?	N/A
Reporting			
Quotations presented	**29**	Were participant quotations presented to illustrate the themes/findings? Was each quotation identified? e.g. participant number	Page 18
Data and findings consistent	**30**	Was there consistency between the data presented and the findings?	Page 10
Clarity of major themes	**31**	Were major themes clearly presented in the findings?	Fig.1

Clarity of minor themes	**32**	Is there a description of diverse cases or discussion of minor themes?	N/A

Developed from: Tong A, Sainsbury P, Craig J. Consolidated criteria for reporting qualitative research (COREQ): a 32-item checklist for interviews and focus groups. International Journal for Quality in Health Care. 2007. Volume 19, Number 6: pp. 349 – 357
