# Supplementary material for: The use and application of intensive care unit diaries: An instrumental multiple case study
Source: PLoS One. 2024 Feb 29;19(2):e0298538. doi: 10.1371/journal.pone.0298538 (PMC10903823; doi:10.1371/journal.pone.0298538)
Supplement: S2 Table — (PDF) [file pone.0298538.s002.pdf]

S2. Table Study protocol for Instrumental Multiple Case Study

Data Collection Procedures

1. Name of contact persons for doing fieldwork; different ICUs were identified and agreed to cooperate. The ICUs were identified as A, B, C, and D to maintain anonymity. Contact persons were nursing staff working with follow-up services in the different ICUs, including head nurses.

2. Data collection plan; to start with observations including field notes in each ICU. Calculated time spending in the ICUs was approximately four working shifts per site. Each occasion in the ICU B, C, and D would include an evening shift and thus subsequent morning shift per site. The researcher stayed overnight in a hotel because of the distance to her own home.
3. Expected documentary material to be reviewed when on site.

   If any memorandum (PM) existed about the caring intervention, writing of a diary with inclusion and exclusion criteria and any statements about when to start a diary.
   If the diary use is included in the introduction for new ICU employees.
   If the diary itself includes a standardized introduction about the purpose of writing the diary.
   If an ongoing diary is documented in the online medical journal system at the participant hospital (COSMIC, ICCA, MELINOR)
   If any standardized information about the diary writing exists to patient and family members.
   If any documents exist concerning informed consent about the use of photographs or writing diaries.

If any records of kept diaries exist

4 Focus group interviews with former patients, and their family members from four different ICUs (A, B, C, D) with varied practices concerning diary writing. If it is unfeasible to carry out the focus group interviews in this form due to a long journey and/or anticipated poorer health status of the former patients, the group interview will be carried out at a different hospital that is geographically closer for the patient concerned.

6 Individual interviews with former patients and their family members and post-graduate specialist nurses in intensive care.

7. Unstructured and informal interviews with head nurses, critical care nurses, assistant nurses, anaesthesiologists, patients, family members, and curators in conjunction with the observation study periods.

Data collection Questions to participant nursing staff in the study

How/where are the diaries kept?

How are photographs managed?

Does any follow-up of the diary writing exist?

If any nursing manager exists?

If any responsibility for diary writing exists?

When during the working shift are nursing staff writing in the diary?

What are the nursing staff writing about?

When is the diary opened up during the patients' stay in the ICU?

Which group/s of care staff are writing in the ICU diary?

What are the exclusion and inclusion criteria?

When during the ICU stay are the patients photographed?

How are the photographs included in the diary?

Are nursing staff photographed with the patient?

When is the diary handed over to the patient?

When are the photographs included in the diary?

Joint Focus group questions to former patients, and family members

Do you have photographs in your diary?

How do you experience the photographs?

Do the photographs cover the whole stay in the ICU?

To what extent (if any) do the photographs contribute to a better understanding of your ICU stay?

Do you miss any photographs from any personal milestone during your ICU stay?

Did you write in the diary?

Do you experience the diary positively or negatively?

What do you experience as the most important aspect/s of the diary?

Informal questions to the ICU nursing staff diary group

When did you start working with ICU diaries?

What was the inspiration/idea for this?
