# Supplementary material for: The use and application of intensive care unit diaries: An instrumental multiple case study
Source: PLoS One. 2024 Feb 29;19(2):e0298538. doi: 10.1371/journal.pone.0298538 (PMC10903823; doi:10.1371/journal.pone.0298538)
Supplement: S3 Table — (PDF) [file pone.0298538.s003.pdf]

S 3 Table. Description of focus group interview participants

Focus Group	I	I	I	II	II	II	III	III
Patient	Patient 1	Patient 2	Patient 3	Patient 1	Patient 2	Patient 3	Patient 1	Patient 2
Age	73	72	71	62	50	61	44	58
ICU length of stay in days	40	70	16	30	35	10	14	5
Length of time discharged from ICU when interviewed	11 months	11 months	11 months	14 months	14 months	8 months	7 months	12 months
Reasons for admission to ICU	Replaced Aortic valve and dissecting aortic aneurism	Lymphoma, Guillain-Barrés syndrome	Fall related trauma	Necrotizing fasciitis	Myocardial infarction	Cardiac arrest	Pneumon ia	Dissecting aortic aneurism
Family member	Spouse 1	Spouse 2	Spouse 3	-	-	-	Spouse 1	Spouse 2
